# Impact of COVID 19 Pandemic and Big Data on China's International Trade: Challenges and Countermeasures

**DOI:** 10.3389/fpubh.2022.888335

**Published:** 2022-06-09

**Authors:** Shuang Zhao

**Affiliations:** Department of Economics, School of Economics, Inner Mongolia Minzu University, Tongliao, China

**Keywords:** psychological approach, nursing, cancer patients, quality of life, big data, blockchain, artificial intelligence

## Abstract

Early 2020 witnessed the coronavirus disease 2019 (COVID-19) pandemic followed by a nationwide lockdown in the whole history for the first time. In this raising dilemma, multiple countries had a serious impact on their international trade, especially during the lockdown. It is also widely accepted that the lives of individuals had been changing ever since the spread of COVID-19. Several other sectors were badly affected during the pandemic. For the above reasons, service industries had a significant impact before and after the pandemic. Based on the data collected, it was identified that the pandemic affected the service industries, enterprises, and other organizations that contribute to the economic growth of the nation. It was also found that the pandemic has adversely impacted private and public enterprises. In addition, the study examined the impact of COVID-19 on China's international trade using artificial intelligence and blockchain technology. Another objective of the article is to examine the impact of big data on China's international trade. The study suggests upgrading the trading policies of China to deal with the challenges being faced in the trading industry.

## Introduction

The unexpected outbreak of coronavirus disease 2019 (COVID-19) in the year 2020 caused several changes in the social and economic lives of the people. To slow down the spread of COVID-19, several businesses and enterprises have decided to impose restrictions on their economic activities. Almost every country in the world declared a nationwide lockdown, banned entry of people from various countries, and imposed social distancing norms (1, 2). Particularly, strict restrictions were followed all around the world. However, these restrictions were lifted gradually. Even the economic activities were started and contributed to the economic growth of the nation. The Gross Domestic Product of China started increasing after the first wave. Though the second wave hit many countries, the learnings and experiences of the first wave allowed people to manage their social and economic activities in the second pandemic (3). The social, political, and economic activities of the countries were badly affected as a result of the pandemic outbreak. The majority of the businesses adopted strict measures, such as wearing masks, following quarantine, and social distancing norms, to curb the spread of the pandemic (4). The economic growth of the country suffered a huge shock, which was transferred to the traders spread across the world. In short, the international trading chain was hit hard, disrupting the supply chain management, reducing the global production, cross-border investment, and trade. China was the first country affected by the pandemic, due to which exports, imports, and trade faced uncertainty.

Several studies have been conducted to investigate the impact of a pandemic on China's international trade. For this, the studies analyzed the data collected from January 2019 to September 2020 on trading. Results of the studies stated that the COVID-19 pandemic predominantly affected trading at the international level at various levels (5). The scales of production, scales of import, scales of export, and the supply of products all slowed down, especially after the pandemic outbreak. There was a decline in the operations across the industries. Due to the decrease in demand for the product during the pandemic, the trading industry had to experience collateral damage, particularly after COVID-19. In other words, the COVID-19 outbreak has negatively affected trading. It has even changed society and even the lives of people. However, it is hoped that these negative effects of the pandemic on international trade will reduce after the first pandemic (6).

During the pandemic, the restrictions laid for trading adversely damaged the international trading system. The growth of international trading began to slow down as a consequence of the destructive effects of the pandemic. In the year 2019, the growth of trade was identified to be sluggish. The pandemic had a negative effect on global trade and made it even more sluggish than ever before. Since the service industry of China is heterogeneous in nature, the effect of the outbreak on the industry was quite different. China is popularly known as the best country for international trading, and hence they started using the internet and big data operations in promoting the trade of the industries (7–11). Big data, generally, uses technology to conduct deep research and analysis (7–9, 12–14). Therefore, the current study also tries to examine the impact of big data on China's international trade, as big data exert impact on the trading enterprises. This article follows the layout given below. LITERATURE REVIEW Section presents the examination of the available literature review. METHODOLOGY Section discusses the methodology adopted in the study and the ways to gather data. RESULTS AND DISCUSSION Section presents the prominent findings derived from the study, followed by a discussion. CONCLUSION Section presents the conclusion of the article.

## Literature Review

An ever-increasing body of literature examines the impact of COVID-19 in those countries that perform international trading and finds that the rapid spread of the outbreak gravely affected the social, political, and economic activities of a country (15). Due to the strict lockdown measures, such as following social distancing norms, banning people from entering the countries, and following quarantine measures, the movement of people from one place to another was reduced. All the educational institutions, workplaces, and tourist spots were closed for a long period of time. The number of cases and deaths was increasing rapidly and as a result, work forces were reduced within the industries. COVID-19 was identified as one of the prolonged diseases and thus it reduced the human resources. For these above reasons, supply chain management was hit hard, which led to a reduction in the supply of goods. The trading curve of China moved upward and much steeper (16). The COVID-19 pandemic negatively affected the trading countries as their lockdown measures interrupted the transportation sector. When the transportation sectors were disrupted, the cost of exporting and importing doubled.

Several studies have put forward that COVID-19 has poorly scaled down the production, supply, and trading, thereby affecting the international trading of the country. However, the degree of damage would differ from one industry to another, based on their heterogeneity (17). These studies also proposed that those industries that supply essential products would not find it difficult to trade their products and will have less damage than industries that produce non-essential services. The essential services include medical products and food, while non-essential products include machines, electronics, and automobiles (17). The supply of essential products was not restricted as it was a universal need. Hence, the manufacturers of the essential products were not affected due to the lockdown measures. Hence, these countries that supplied essential products were able to supply these products to the needy even during the lockdown period. Moreover, shutting down the factories was not exceptionally applicable to those industries (17).

Despite all these damages, most of the industries started operating remotely, which partially reduced the negative impacts of the pandemic on the industries. With the help of information technology and telecommunication services, industries could successfully move to remote work and thereby reduce the shocks in the supply of goods and services, especially in the manufacturing services (18). Most countries are successful in maintaining their economic activities by the means of advanced information technology (IT) and telecommunication services. Indeed, these services could largely mitigate the negative effects of the pandemic on international trade. Surprisingly, these new advancements also increased the productivity and efficiency of the scale of production, which helped the manufacturers to increase scales of production. By doing so, the industries could increase their trading performance (19). Most importantly, a lot of industries moved to remote work from on-site production services after analyzing their success rate. On the other hand, those industries that continue to work on-site observed a decrease in international trading, despite adapting to the advanced technology services. Industries, such as footwear, textile, leather, retail, machinery, and transportation sectors, find it extremely difficult to increase their scales of production when they do not shift from operating on-site to remote (20, 21). Few other studies also pointed out the fact that the production of goods and services generally demands the presence of an individual and it is considered critical. Of all the manufacturing jobs, only 22% of them can be done remotely, while others would require the presence of a person.

Countries that are importing products or services will be mainly affected in terms of cost. The lockdown measures will be a hindrance to the people's earnings, and thus their demand for the products will naturally come down. As the earnings of the people will be affected due to the strict lockdown norms, the governments will cut down the demands to meet the needs of the people who need support. This can lead to shrinkage of the demands, resulting in supply shocks (22). Most importantly, the spread of disease in a rapid manner discourages people from visiting supermarkets and other essential outlets, which can also lead to a shrinkage in demand. This demand shock negatively impacts the industries. A few authors have also examined the impact of the recession on international trade and found that people who are facing such shock spend less on durable products than on non-durable products, as the latter can be deferred (23). Therefore, the demand shock for durable products, such as footwear, machinery, wood products, textiles, rubber, plastics, leather products, glass products, and precious metals, can be much higher.

A large number of enterprises have relatively started using big data technology to seek information/data. As part of economic globalization, many countries have adapted big data to meet specific requirements (24, 25). The current advancements in the field of IT are commonly referred to as big data. As data handling becomes difficult each day due to rapid development in the internet databases, enterprises find it difficult to manage their data regarding exports and imports. In other words, the data/information of trading is inseparable from internet databases (7, 26, 27). To make appropriate use of resources and as a part of economic globalization, organizations utilize big data. In addition, with an intent to enhance the business and derive profits, enterprises make use of big data (7, 8). Thus, this era is called as the era of big data. Examining data using big data technology is considered scientific, logical, and perfect in nature, when compared with traditional enterprise management (9, 12, 13). To improve the quality of a business, SMEs make use of big data to make an accurate and quick decisions in the need of the hour (7, 27). The literature review shows that the use of big data in the business will enhance the whole process of business, and for this reason, SMEs are considering adopting big data to businesses (12). The use of big data technologies will help SMEs in deriving market value. The best way to deal with the challenges in business is to adopt big data in SMEs (9, 12). However, researchers have shifted their focus to big data, on discovering the benefits of big data.

**Figure 1:**
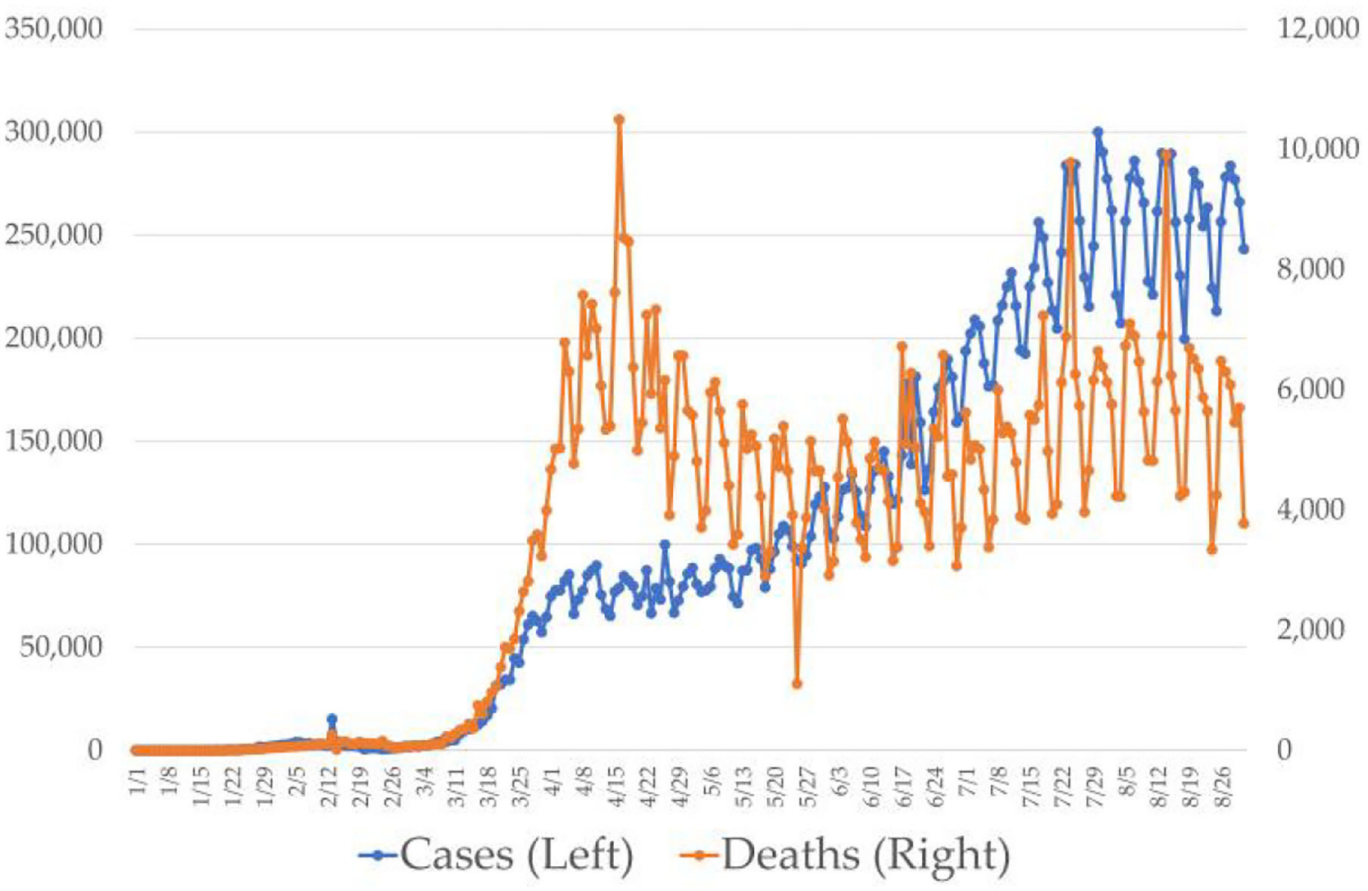
Coronavirus diseases 2019 (COVID-19) cases and deaths recorded during the first wave (*X*-axis, number of deaths; *Y*-axis, cases reported on a daily basis).

A series of recent studies have indicated that multinational organizations are buying big data. Even startups are purchasing big data technologies to understand the new market, new clients, new business, and so forth. Big data will also help enterprises to cut down on their costs of business and enter the market. Transforming the big data to business applications will cost them real difficulty. Investing capital in big data hardware and software is high. Few other studies revealed that technology adaptation, financial innovations, and big data are regarded as the strong pillars of enterprises (23). Organizations that use big data can increase their productivity and growth, which will improve the performance of enterprises. It will also help organizations to understand the behavior of the market. There exists a considerable body of literature on the importance of data within companies. Data are regarded as important asset for enterprises. Large industries across the world are exploring different ways to tackle the data, which are usually called big data (26). Figure 1 shows the COVID-19 cases and deaths recorded during the first wave.

Few other studies stressed that enterprises could use big data technology to facilitate trading. Enterprises can come up with different strategies to enhance international trade. As per the international trade theory, economic exchanges between the countries will reflect their competitive spirit in their economic activities (7, 9, 12). Profit being the ultimate aim of any business, enterprises ensure to meet the needs of customers, thus adding value to the business. Efficient and productive business always help the industries to understand the market and improve the commercial value of the products. It will also enrich the scope of the market by providing innovative ideas for the enterprises to operate more successfully. Recent studies have concluded that the present era is popularly known as the era of big data and economic globalization (13). Researchers have come up with different trading models that can increase profit. One such model is to utilize the internet database and then make significant decisions on trading products at the international level. It is also one of the prominent ways to improve the strength of enterprises. Big data also helps enterprises to understand the international market before entering the global market (8). This technology provides good protection to enterprises. Hence, the organizations will be able to participate in international trade and produce additional products. It can therefore be concluded that big data has advantages and disadvantages. From the analysis of the existing literature, the study proposes the following hypotheses:

*H1: There is a negative relationship between the COVID-19 pandemic and International Trade*.

*H2: There is a positive relationship between the COIVD-19 pandemic and big data technology*.

*H3: There is a positive relationship between big data and international trade*.

*H4: Big data increases the growth and development of enterprises, even during the pandemic*.

*H5: Technology adaptation and big data technologies will increase the international trade even during the COVID-19 pandemic*.

## Methodology

The current study mainly concentrates on examining the impact of COVID-19 and big data on China's international trade. In addition, it also examines the relationship between COVID-19 and international trade; and the COIVD-19 pandemic and big data technology; adopting big data technologies from the perspective of international trading. The analysis of the current study has been conducted through secondary literature and primary analysis as well. The study has developed a hypothesis based on the analysis and review of the secondary literature. The formulated hypothesis is tested using a structured questionnaire. Thus, the current study gathers all the required data using a questionnaire and hence the study is descriptive. Using the questionnaire's results, the researcher will evaluate the hypothesis. This method involves describing the objectives, defining the population, selecting the sample, and later interpreting the data and results. The population is a complete set of individuals who are bound together by certain common characteristics. The population is of two types: target and accessible. The target population consists of those whom the researcher wishes to generalize the findings of the study. In the current study, the total population includes enterprises that participate in international trading in China. From the total population, the study derives the required sample. The term sample includes the individuals selected to participate in the study. These selected participants are popularly known as respondents/subjects of research. Sampling methods are of two types: probability/random sampling and non-probability. To carry out the research, this study employs a random sampling method. At times, researchers also tend to use purposive sampling to obtain required data from certain areas of research.

To fulfill the objectives of the study, the study selects a sample consisting of 5,210 enterprises located in China. This study focused on those international trading enterprises in China that were relatively easy to connect using an electronic channel. Prior to the administration of the questionnaire prepared, consent from the enterprises was imitated as the first step. Only after the participant's acknowledgment, the questionnaire link was distributed to the selected sample. Out of 5,210 samples, approximately 4,500 of them acknowledged and agreed to participate in the study. So, in total, 4,500 SMEs sent their prior consent, out of which only 3,000 were considered valid. Based on the analysis of the secondary literature, the researcher prepared a structured questionnaire, which addressed the factors, such as the relationship between COVID-19 and international trade, the COIVD-19 pandemic and big data technology, adoption of big data technologies, and challenges in the international trading and its countermeasures from the perspective of international trading. This structured questionnaire will allow the selected respondents to share their views, opinions, beliefs, attitudes, and values on the research topic. The data were collected from October 2020 to December 2020 and a sample of 3,000 was considered along with their responses. Table 1 presents the technical details of the research. In total, 2,000 responses were collected back and after the screening process, data of 1,500 respondents were finalized and considered for further analysis. Both primary and secondary data are collected to find out the results. The primary data are collected using the structured questionnaire designed to collect the required data from the participants. The collected data will help the researcher to examine the relationship between COVID-19 and international trade, the COIVD-19 pandemic and big data technology, adopting big data technologies, and challenges in the international trading and its countermeasures from the perspective of international trading. The researcher collects the secondary data required for the study from research articles and other journals. Finally, all gathered information was collected and analyzed to answer the research purpose.

**Table 1:** Research details.

**Headers**	**Comments**
Sector	Enterprises/Industries involved in International Trading
Location	China
Methodology	Structured questionnaire
Sampling technique	Technique of random sampling
Selected respondents	2,000 (1,500)
Period of data collection	October 2020 to December 2020

**Figure 2:**
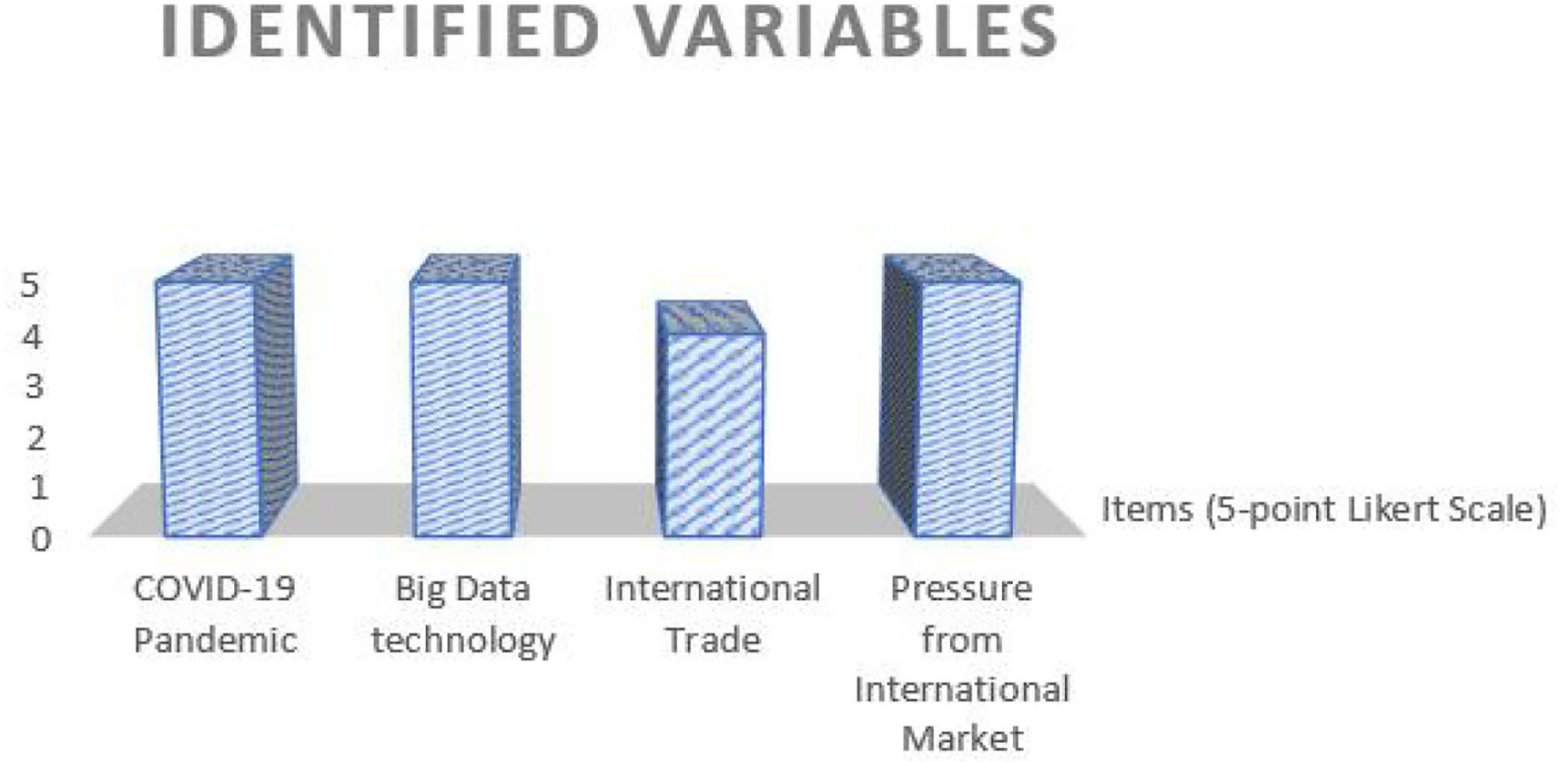
Graphical representation of the identified variables (*X*-axis, variables identified in the study; *Y*-axis, rating scale).

The present study identifies different variables, such as big data technology, COVID-19 pandemic, international trade, and pressure from international market along with the measurement model. This model is presented in Table 2 and its graphical representation is presented in Figure 2.

**Table 2:** Variables of the study.

**Name of the variable**	**Variable definition**	**Items** **(5-point Likert Scale)**
COVID-19 pandemic	This variable helps the researchers to understand the market conditions, analyse the essential needs of the customers across the industries, and propose a new way of remote operations/work leading toward economic globalization	5
Big Data technology	Big Data refers to massive volumes of data, which expands the business with new opportunities and challenges, even at the international level. Adopting innovative technological advancements within the industries will help them to enhance their services to customers, improve supply chain linkage, and strengthen their knowledge of market and trade relationships	5
International trade	Understand the concept of international trade, which will help the researcher to examine the impact of COVID-19 and Big Data on China's International Trade	4
Pressure from international market	Understand the International market, which will help the researcher to examine the impact of COVID-19 and Big Data on China's International Trade. It also helps the researcher to understand the challenges faced by the industries with regard to trading, during the pandemic times and suggest the countermeasures	5

To analyze the data collected, the researcher employs Statistical Package for the Social Science (SPSS) and analysis of moment structure (AMOS) 26.0 version. To study the relationship between the variables identified, the study adopts structural equation modeling (SEM). Furthermore, the selected enterprises/industries will be able to provide accurate and precise information on the questions covered in the questionnaires related to the research topic. As the SEM method is highly resilient and efficient in modern times, the researcher decided to use the same. This method will help the researcher to examine the relationship between the variables identified by testing the proposed hypothesis. This method also focuses on the appropriateness of the results and hence was taken into consideration.

Although the owners of enterprises were reluctant to share the details of trading, such as their performance during the pandemic, challenges faced during pandemic times to trade the services/products, adopting big data technologies for smooth trading, and their point of view on the international market, from the perspective of international trading sustainability initially, later, they understood the need to evaluate the challenges faced by the enterprises in terms of trading, especially during the pandemic times, which helped the researcher to record the accurate data. Past researchers also tried to measure the impact of COVID-19 and big data on international trading, which were in line with the opinions of the respondents, marked on a 5-point Likert scale, scaling from extremely poor to extremely good. The study also recorded responses from the participants on innovative ways to manage trading among China's enterprises from the perspective of profit and development. Lastly, the study also recorded the responses of the respondents on adapting to innovative technologies and big data within enterprises that will enhance international trading on a 5-point Likert scale.

## Results and Discussion

**Figure 3:**
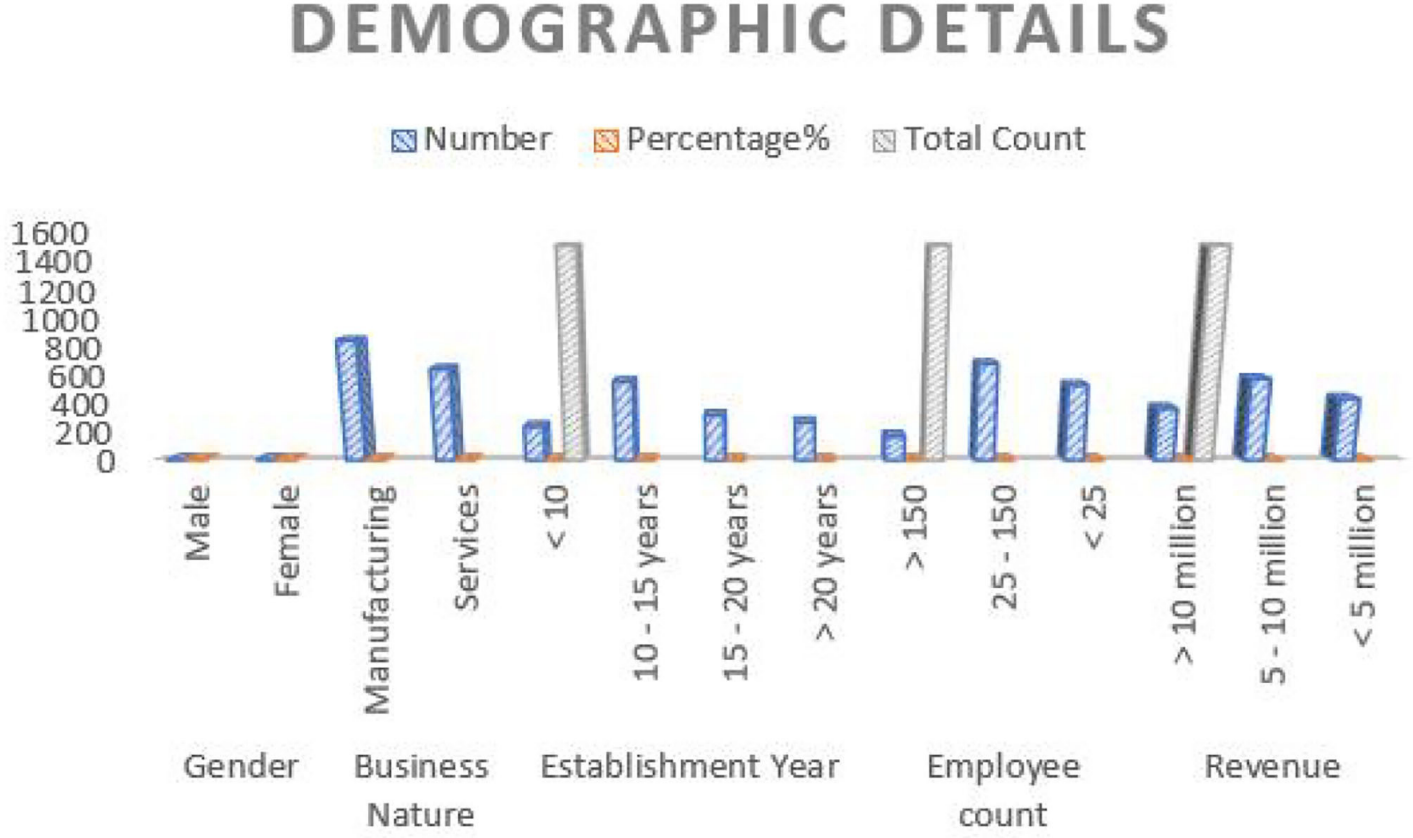
Graphical representation of demographic details (*X*-axis, demographic variables; *Y*-axis, number of respondents).

The current study confirmed the following findings using artificial intelligence and blockchain technology. Table 3 records the demographic details of the respondents, who participated in the structured questionnaires. Figure 3 provides a graphical representation of demographic details. When analyzing the data of the enterprises, it was understood that 64.3% of them are men and 36.3% of them are women, spread across industries, such as manufacturing and services, essential products, such as food and medical manufacturing enterprises, units of footwear, leather, textiles, machinery, and transportation services. About 55.05% of the respondents come under the essential products manufacturing units and 46.21% of them belong to service units, such as beauty parlors, book shops, and transportation services. Similarly, 40.31% of the enterprises were established for almost more than 10 years and come in between 15 years, 22.18% of the enterprises were established for more than 15–20 years, 19.61% of the enterprises were established for more than 20 years, and least 18.4% of them were established for less than 10 years. With regard to employee count, 49.21% of them disclosed a high level of engagement in operations, 39.41% disclosed a mediating level of employee engagement, and 13.01% exhibited low levels of employee engagement. Lastly, about the employee revenue, 42.60% of them earned almost 5–10 million in a year, followed by 32.23% (<5 million) and 27.21% (<10 million).

**Table 3:** Demographic details of the respondents.

**Details**	**Number**	**Percentage%**	**Total count**
**Gender**			
Male	950 (M)	64.3%	1,500
Female	550 (F)	36.3%	
**Business nature**			
Manufacturing	850	55.05%	1,500
Services	650	46.21%	
**Establishment year**			
<10	242	18.4%	1,500
10–15 years	561	40.31%	
15–20 years	322	22.18%	
>20 years	272	19.61%	
**Employee count**			
>150	172	13.01%	1,500
25–150	687	49.21%	
<25	536	39.41%	
**Revenue**			
>10 million	371	27.21%	1,500
5–10 million	582	42.60%	
<5 million	442	32.23%	

**Figure 4:**
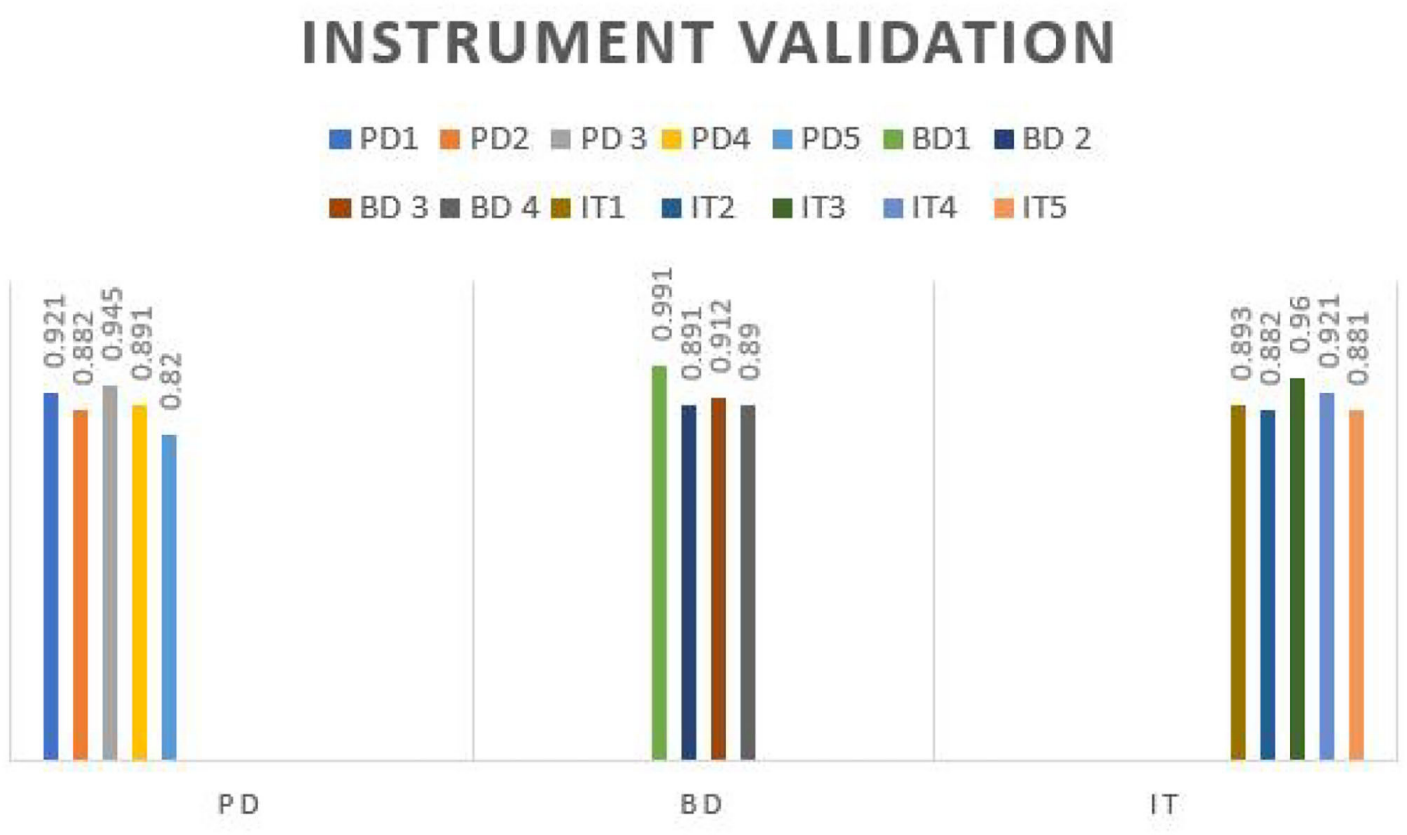
Graphical representation of validating the instrument (*X*-axis, variables identified; *Y*-axis, instrument validation).

The validation of the instrument used in the study is recorded in Table 4. From the table, it can be understood that the skewness test range results suggest that variables identified in the study—big data technology, COVID-19 pandemic, international trade, and pressure from international market are equally distributed. The extreme loading values presented in the table state that the instrument used is highly reliable and valid, which is calculated as 0.7 in terms of implementation. Moreover, the results presented in Table 4 also confirm the absence of multicollinearity in the data gathered. Figure 4 presents the graphical representation of validating the instrument used in the study.

**Table 4:** Validating the instrument used in the study.

	**PD**	**BD**	**IT**
PD1	0.921		
PD2	0.882		
PD 3	0.945		
PD4	0.891		
PD5	0.820		
BD1		0.991	
BD 2		0.891	
BD 3		0.912	
BD 4		0.890	
IT1			0.893
IT2			0.882
IT3			0.960
IT4			0.921
IT5			0.881

The result of the empirical study shows that implementing big data technology within the enterprises will be the best way to manage international trading, during the pandemic times, as big data plays a crucial role from the perspective of industry sustainability, as the coefficient value seems to be higher than the cut-off value. Similarly, the results associated with variable COVID-19 pandemic also confirmed that they seem to significantly impact the smooth functioning of the trading, due to the financial pressure these industries face in China. It was also found that the international trading market will slow down, especially when advanced technologies are not implemented, which are in line with enterprise sustainability from the perspective of the pandemic. All the identified variables seem to be efficient and hence the results can be considered reliable. The findings of the study also disclosed that there are no reliability and validity issue about the identified constructs.

**Figure 5:**
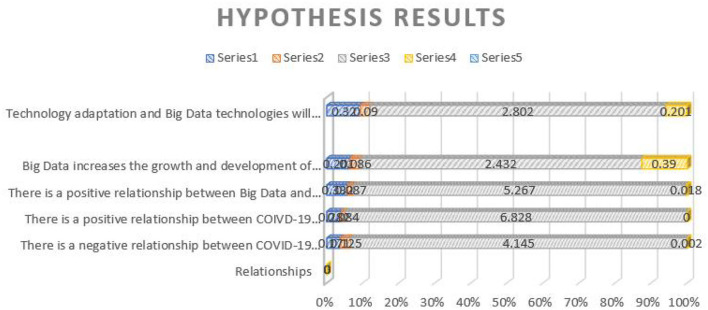
Graphical representation of results of hypothesis testing (*X*-axis, hypothesis testing results; *Y*-axis, hypothesis stated).

Moreover, the study identified several challenges, such as facing extreme financial pressure, pressure for the industries or enterprises to explore the international market, the pressure of ordering goods/services, the pressure of transferring goods and services, and others. During the pandemic times, industries faced operating difficulties as there were strict lockdown measures, which turned the lives of people upside down. People have started spending less on durable products than on non-durable products. For these reasons, the economic activities during the pandemic were stringent. Earnings of the workers were less, and hence people were skeptical about spending on non-essential items. Even the transportation services were banned from one place to another as the spread of the disease was rapid. All these factors reduced the export and import of goods/services from one country to another. Hence, it can be stated that the pandemic negatively affected international trading, thereby lowering the economic growth of the country. As a countermeasure, these enterprises can implement big data technologies or advanced ITs, which will help them carry out the operation remotely. Past studies also confirmed that new technologies in the field of IT were extremely optimistic about the pandemic situation. It was equally important to understand the international market and develop international trade, especially during the pandemic times. Generally speaking, businesses faced a huge decline in their profits when the pandemic hit hard, which also badly impacted international business. In addition, the study presented the results in measurement models, including all the identified various variables. Table 5 presents the measurement model results of the study, which indicate that there exists a relationship between the constructs—innovative finance, adopting technology within the SMES, and adapting to big data technologies from the perspective of SME sustainability and big data. Figure 5 represents the results of hypothesis testing.

**Table 5:** Results of hypothesis testing.

**Relationships**	**Path**	**SD**	***t*** **-Value**	***p*** **-Value**
H1: There is a negative relationship between COVID-19 pandemic and International Trade	0.171	0.125	4.145	0.002
H2: There is a positive relationship between COIVD-19 pandemic and Big Data Technology	0.282	0.084	6.828	0
H3: There is a positive relationship between Big Data and International Trade	0.332	0.087	5.267	0.018
H4: Big Data increases the growth and development of enterprises, even during the pandemic	0.201	0.086	2.432	0.39
H5: Technology adaptation and Big Data technologies will increase the international trade even during the COVID-19 pandemic	0.32	0.09	2.802	0.201

The study found that the hypothesis proposed in the study has a high impact on enterprises' international trading and sustainability. The results of the study document that there is a negative relationship between the COVID-19 pandemic and international trade; there is a positive relationship between COIVD-19 pandemic and big data technology; there is a positive relationship between big data and international trade; big data increases the growth and development of enterprises, even during the pandemic, and technology adaptation and big data technologies will increase the international trade even during the COVID-19 pandemic. In addition, the study found that the enterprises performing international trading in China are high in number and hence the operations of the enterprises should focus on adapting to innovative technology, such as big data to sustain in the international market. Even big data technologies will create new challenges and opportunities for the enterprises in the contemporary world and enhance trading. Furthermore, there is a positive relationship between adopting technology and international trading, which will expose enterprises to new opportunities and challenges. In summary, big data technologies are essential in enterprise sustainability, even though a pandemic has hardly hit the trading industries.

## Conclusion

The present article has proposed and tested the relationship between the COVID-19 pandemic and big data on China's international trade. The results of the study indicated that there is a negative relationship between the COVID-19 pandemic and international trade; there is a positive relationship between COIVD-19 pandemic and big data technology; there is a positive relationship between big data and international trade; big data increases the growth and development of enterprises, even during the pandemic, and technology adaptation and big data technologies will increase the international trade even during the COVID-19 pandemic. The study also identifies the challenges faced by the industries during pandemic times and thus, as a countermeasure proposed adapting big data technology implementation within the industries to enhance international trade.

## Data Availability Statement

The original contributions presented in the study are included in the article/supplementary material, further inquiries can be directed to the corresponding author.

## Ethics Statement

Ethics review and approval/written informed consent was not required as per local legislation and institutional requirements.

## Author Contributions

The author confirms being the sole contributor of this work and has approved it for publication.

## Conflict of Interest

The author declares that the research was conducted in the absence of any commercial or financial relationships that could be construed as a potential conflict of interest.

## Publisher's Note

All claims expressed in this article are solely those of the authors and do not necessarily represent those of their affiliated organizations, or those of the publisher, the editors and the reviewers. Any product that may be evaluated in this article, or claim that may be made by its manufacturer, is not guaranteed or endorsed by the publisher.
